# Seed germination of *Agave* species as influenced by substrate water potential

**DOI:** 10.1186/0717-6287-47-11

**Published:** 2014-04-01

**Authors:** Hugo M Ramírez-Tobías, Cecilia B Peña-Valdivia, Carlos Trejo, J Rogelio Aguirre R, Humberto Vaquera H

**Affiliations:** Facultad de Agronomía, Universidad Autónoma de San Luis Potosí, Carretera San Luis Potosí —, Ejido Palma de la Cruz, Soledad de Graciano Sánchez, Matehuala km 14.5, San Luis Potosí, 78321 México; Botánica, Campus Montecillo, Colegio de Postgraduados, Carretera México-Texcoco km 36.5, Texcoco, Estado de México CP 56230 México; Instituto de Investigación de Zonas Desérticas, Universidad Autónoma de San Luis Potosí, Altaír 200, Fraccionamiento del Llano, San Luis Potosí SLP CP 78377 México; Estadística, Campus Montecillo, Colegio de Postgraduados, Carretera México-Texcoco km 36.5, Texcoco, Estado de México CP 56230 México

**Keywords:** Base water potential, CAM, Drought, Germination, Hydrotime model, Maguey

## Abstract

**Background:**

Plants of *Agave* spp. perform Crassulacean acid metabolism (CAM) and are highly drought-tolerant, but little is known concerning seed germination under low water availability. The aim of this study was to assess the effect of substrate water potential (Ψ_W_) on seed germination and contrast hydrotime parameters of seven valuable and commercially-important *Agave* species from different geographical distributions and climatic regions of Mexico. Our hypothesis was that seed germination of *Agave* species is not affected by low water availability independently of seed biomass and the climate of their distribution area.

**Results:**

Seed germination (at 25°C and in the dark) between 85 and 100% for all species occurred within 80–180 h at -0.03 MPa and 250–430 h at -1.0 MPa. Seed germination at -1.5 MPa declined to less than 50% (p < 0.05) for *A. asperrima* and *A. cupreata* but did not change significantly for *A. americana* var. *marginata*, *A. lechuguilla* and *A. striata*, although they showed the lowest mean base water potential (-2.01 to -2.64 MPa). Seed germination of 40% *Agave* species, from arid and semi-arid climates in this study, was not affected by the lower Ψ_W_.

**Conclusion:**

Germination of seeds of *Agave* species is moderately affected by low water availability, is partially dependent of their ecological distribution, and is independent of seed mass.

## Background

Approximately 74 *Agave* species and 28 intraspecific taxa have been used in Mexico as human food, fodder, raw material for fermented beverages and to obtain fibers; at least 48 of these species are currently used to produce spirits called “tequila”, “mezcal”, “bacanora” and other distilled with economic importance [1]. The agave plant is described as a monocarpic perennial monocot that produces an inflorescence only once towards the end of its long life cycle. Each plant produces several hundred seeds and it has been demonstrated for a number of *Agave* species and genotypes that a large proportion of these seeds are viable [1, 2]. *Agave* plants reproduce both sexually and asexually, but they commonly propagate asexually via rhizomes and bulbils in the wild [3]; this propagation favours successful seedling establishment by initial dependence on the mother plant.

*Agave* species are Crassulacean acid metabolism (CAM) plants distributed throughout a wide variety of environments, although a large number of them are found in mesophyte communities [4]; moreover, they tolerate high heat and dry conditions and efficiently produce a large biomass in these environments [5], where few commercial crops can grow. It has also been shown that after germination under optimal conditions, seedlings of *A. salmiana* can grow on a substrate with a water potential (Ψ_W_) of -2.5 MPa [6] and young and adult plants of *A. salmiana* and *A. angustifolia* ssp. *tequilana* maintain active photosynthetic pathway after six months without water, and even throughout the whole dry season [7, 8]. It has also been observed that root and leaf growth of young *A. salmiana* plants is affected by frequent irrigation [8]. In addition, the diversity of *Agave* species and environments where they are distributed show an array of specific physiological responses associated with the climate where these plants naturally grow. This evidence suggests that agave seed germination, in addition to other physiological processes, might develop properly in conditions which are suboptimal for other species.

Germination is affected by intrinsic (species and seed size) and environmental (temperature and water availability) factors; however, the seed germination of species adapted to dry environments and cultivars selected for drought tolerance are less affected by low substrate Ψ_W_ than those adapted to wet environments or classified as drought-sensitive [9, 10]. The hydrotime model has been used to quantify the effect of substrate Ψ_W_ on seed germination. The parameters that describe this model allow quantification of the response of a seed germination population to the substrate Ψ_W_ and its biological variation [11]‒[13] and are also useful in understanding some of the ecological relationships between plant species [9, 14, 15].

The aim of this study was to assess the effect of substrate Ψ_W_ on seed germination and contrast hydrotime parameters of seven valuable and commercially-important *Agave* species, with a long history of human usage and from different geographical distributions and climatic regions of Mexico. Our hypothesis was that seed germination of *Agave* species is not affected by low water availability independently of seed biomass and the climate of their distribution area.

## Results

### Seed water uptake

**Figure 1:**
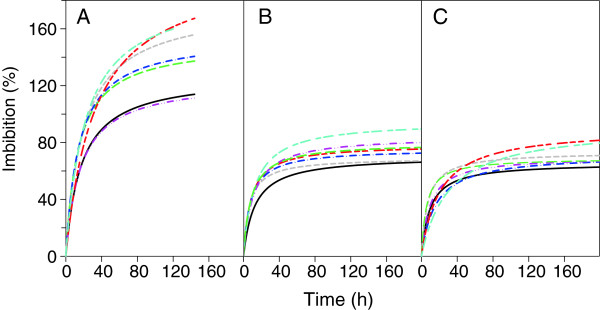
**Calculated time-courses of cumulative seed water uptake of** ***Agave*** **spp. at 25°C, in the dark, and in substrate water potentials of -0.03 (A), -1.0 (B) and -1.5 MPa (C).** A. *americana* var. *marginata* (black line), *A. asperrima* (red line), *A. cupreata* (green line), *A. duranguensis* (gray line), *A. lechuguilla* (dark blue line), *A. salmiana* (pink line) and *A. striata* (light blue line); n = 50.

**Figure 2:**
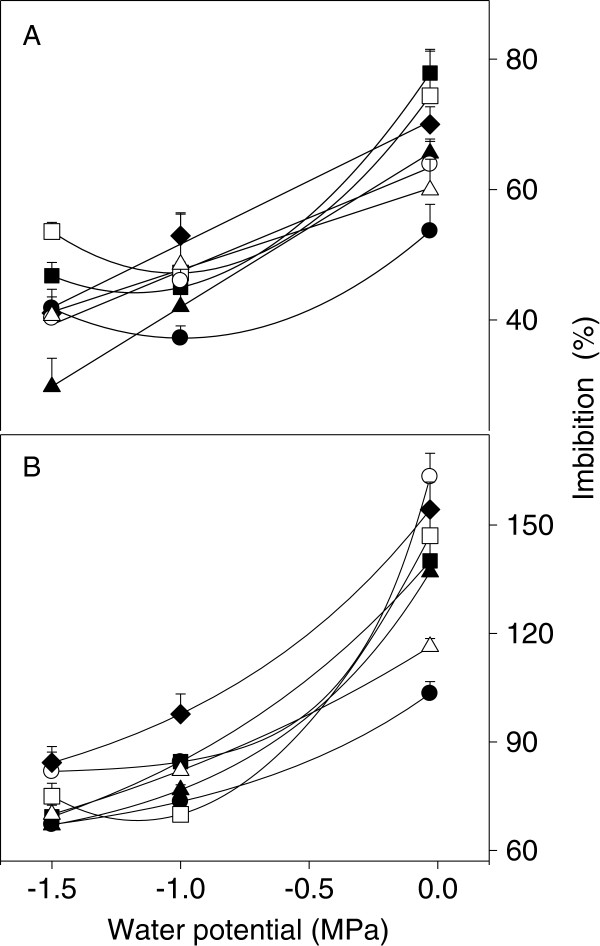
**Initial seed water uptake (after the first 12 h) (A) and maximum seed water uptake (B) of** ***Agave*** **spp. during germination in different substrate water potentials, in the dark and at 25°C.** *A. americana* var. *marginata* **●**, *A. asperrima* **○**, *A. cupreata* ■, *A. duranguensis* **◻**, *A. lechuguilla* ▲, *A. salmiana* Δ and *A. striata* ♦, *n* = 50.

Seed water uptake with maximum water availability varied significantly (p < 0.05) among species (Figure 1). Imbibition by *A. cupreata* and *A. duranguensis* was 20% higher than for *A. americana* and *A. salmiana* during first 12 h of germination at -0.03 MPa, at Ψ_W_ lower than -0.03 MPa decreased significantly (between 42% and 67%) for all species, and with the exception of *A. americana*, *A. cupreata* and *A. duranguensis* decreased linearly. Also, of these three species, initial seed water uptake at -1.5 was not significantly different (p > 0.05) than at -1.0 MPa (Figure 2A).

Differences and similarities in seed water uptake changed with time and with substrate Ψ_W_. Maximum seed water uptake of *A. asperrima* and *A. striata* was significantly higher (165%) than for the other five species and strongly contrasted with that for *A. americana* and *A. salmiana*, which had the lowest water uptake (100 and 115%) (p < 0.05) of all seven species at a Ψ_W_ of -0.03 MPa (Figures 1A and 2B). The initial accelerated water uptake up to maximum germination, was on average 43% lower (*p* < 0.05) at both low Ψ_W_ (40 h) than at -0.03 MPa (70 h) (Figure 1A–C). Maximum seed water uptake of all *Agave* species reduced significantly in a non-linear fashion (50% on average) with lower Ψ_W_ (Figure 2B) and the large differences observed at -0.03 MPa among species almost disappeared at -1.0 and -1.5 MPa (Figure 1A–C). In general, maximum seed water uptake of *A. striata* and *A. asperrima* was among the highest at all three Ψ_W_, whereas that of *A. americana* was among the lowest. Other differences in seed water uptake were that only *A. asperrima* reached similar (p > 0.05) maximum seed water uptake at -1.0 MPa and -1.5 MPa; and only *A. duranguensis* reached a higher (p < 0.05) maximum seed water uptake at -1.5 MPa than at -1.0 MPa (Figure 2B).

### Radicle emergence

**Figure 3:**
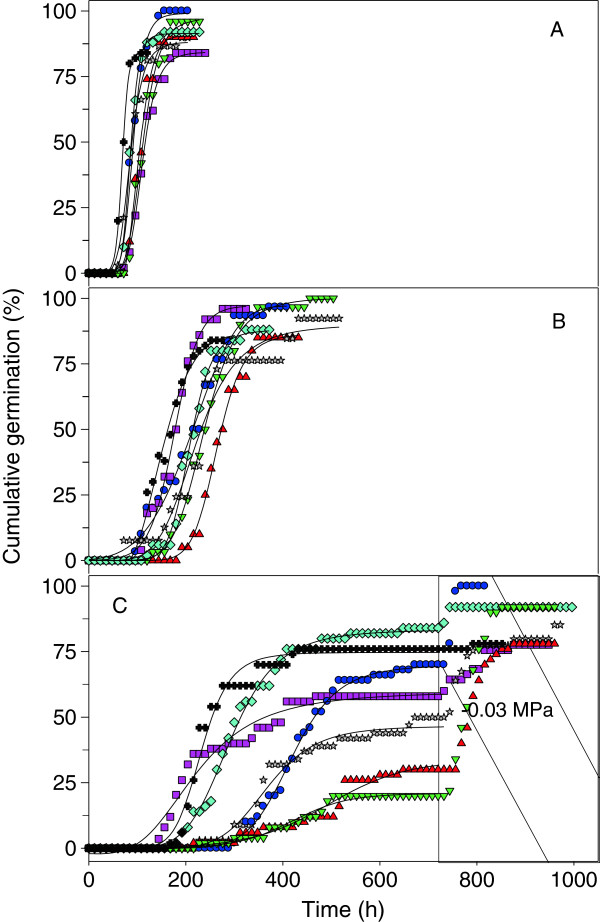
**Time-course of cumulative germination of** ***Agave*** **spp. seeds in substrate at -0.03 MPa (A), -1.0 MPa (B), and -1.5 MPa followed by -0.03 MPa (shaded area; C), in the dark and at 25°C.** *A. americana* var. *marginata* (crosses), *A. asperrima* (red triangles), *A. cupreata* (green triangles), *A. duranguensis* (gray stars), *A. lechuguilla* (blue circles), *A. salmiana* (pink squares) and *A. striata* (blue diamonds), n = 50.

All seven *Agave* species lacked significant seed dormancy and mean cumulative germination (radicle emergence) at -0.03 MPa was 84–100% in 80 to 180 h, depending on the species (Figure 3A). On average, germination at -0.03 MPa started at 72 h and significantly increased almost each 12 h, up to 120 h. Maximum cumulative germination was similar at Ψ_W_ of -1.0 MPa and at -0.03 MPa, but the time taken to reach the maximum increased more than two-fold (Figure 3A-B). On average, germination at -1.0 MPa started after 132 h and significantly rose for 240 h, but the cumulative germination increase was significantly each 12 or 24 h, and was slower than at -0.03 MPa. In contrast, a Ψ_W_ of -1.5 MPa reduced on average 50% the maximum cumulative germination of all species in comparison with -0.03 and -1.0 MPa, except for *A. striata* and *A. americana* (Figure 3C). Germination at -1.5 MPa started after 209 h, but was significantly different from the starting level until 228 h (almost zero), this Ψ_W_ caused significantly rise of germination at 12, 24 and 36 h periods, but this rise was slower than at -1.0 and -0.03 MPa.

**Figure 4:**
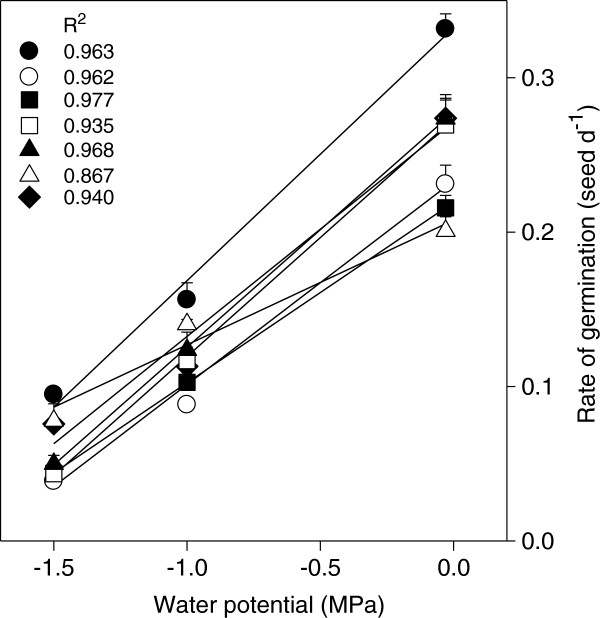
**Effect of the substrate water potential on the rate of** ***Agave*** **spp. seed germination (1/t**_**50**_ **).** *A. americana* var. *marginata* **●**, *A. asperrima* ○, *A. cupreata* ■, *A. duranguensis* □, *A. lechuguilla* ▲, *A. salmiana* Δ and *A. striata* ♦. Lines indicate the water potential effect on the evaluated attribute, predicted by linear functions. The vertical bars indicate one standard error, *n* = 50.

The rate of germination at Ψ_W_ between -0.03 and -1.5 MPa decreased linearly (p < 0.05) for all species (on average from 0.26 to 0.06 seeds d^-1^), however, the decrease in *A. salmiana* was the lowest (0.2 to 0.1 d^-1^; Figure 4). The hydrotime parameters of *A. salmiana* were notable among all species as it had the highest θ_H_ and σ_Ψb_ and the lowest Ψ_b(50)_. All species showed a relatively similar θ_H_ between 140 and 190 (Table 1), except for *A. salmiana*.

**Table 1:** **Parameters of the hydrotime model for seed germination of seven** ***Agave*** **species under three substrate water potentials (-0.03, -1.0 and -1.5 MPa)**

Species	θ _H_ (MPa h)	Ψ _b(50)_ (MPa)	σ _Ψb_ (MPa)	r ^2^
*A. americana* var. *marginata*	142	-2.01	0.44	0.823
*A. asperrima*	158	-1.58	0.37	0.881
*A. cupreata*	190	-1.82	0.46	0.814
*A. duranguensis*	137	-1.68	0.58	0.775
*A. lechuguilla*	153	-1.84	0.36	0.892
*A. salmiana*	282	-2.64	0.81	0.768
*A. striata*	176	-2.02	0.41	0.829

θ_H_: hydrotime constant; Ψ_b(50)_: base water potential; σ_Ψb_: standard deviation of Ψ_b(50)_; *r* ^*2*^: determination coefficient.

### Recovery of germination

A large proportion (20-80%) of seeds of several species did not germinate after a relatively long period (732 h) at Ψ_W_ of -1.5 MPa. However, germination continued after 12–48 h when these seeds were transferred to a substrate with a Ψ_W_ of -0.03 MPa*.* Thus, *A. cupreata*, *A. striata*, *A. duranguensis* and *A. lechuguilla* reached the second final maximum cumulative germination (85-100%) similar to that at -1.0 and -0.03 MPa (p > 0.05). Likewise, germination of the other three species also increased up to 80% after being transferred to the highly hydrated substrate (Figure 3C). Under these conditions, most *Agave* species tolerated slow seed water uptake and survived for more than 30 d with 70% hydration without damage.

## Discussion

### Seed water uptake

Germination phases I and II for the seven species (Figure 1) were similar to those of dried mature seeds of several *A. salmiana* genotypes [2]. Differences of initial seed water uptake in phase I under maximum water availability (Figure 2A) show that *A. cupreata*, which originates from a sub-humid climate (838 mm annual mean precipitation; Table 2), imbibed more water than species from arid and semi-arid climates (340–361 mm of precipitation) i.e. *A. lechuguilla*, *A. americana* and *A. salmiana*. However, this response was not common to all *Agave* species but was the case for *A. striata*, with a high water uptake during phase I of germination under the three substrate Ψ_W_, it possibly can happen as an adaptation to arid habitats (287 mm of precipitation).

**Table 2:** ***Agave*** **species used in this study and characteristics of collection sites in Mexico** ^**A**^

Subgenera	Species	Collection site	Reproduction	Climate (MAP)†	Type of vegetation
*Agave*	*A. americana* var. *marginata*	San Luis Potosí, SLP	Rhizomatous offshoots and seeds	Various (580)	Artificial habitats and ornamental
	*A. asperrima*	Cerritos, San Luis Potosí	Rhizomatous offshoots and seeds	BS_1_kw (450)	Piedmont scrub and xerophytic shrubland
	*A. duranguensis*	Guanajuato, Gto.	Rhizomatous offshoots and seeds	BS_0_kw(e) (484)	Xerophytic shrubland, open pine, and oak forest
	*A. cupreata*	Chilapa, Guerrero	Seeds	A(C)w_1_(w)(i’)g (838)	Pine and oak forest, pastureland and palm grove
	*A. salmiana*	La Mantequilla, San Luis Potosí	Rhizomatous offshoots and seeds	BS1hw(e)g (361)	Succulent shrubland and mycrophyllous dry shrubland
*Littaea*	*A. lechuguilla*	Guadalcázar, San Luis Potosí	Rhizomatous offshoots and seeds	BW, BS_0_, BS_1_ and CW_0_ (340)	Xerophytic rosette shrubland
	*A. striata*	Guadalcázar, San Luis Potosí	Seeds and axillary branching	BShw (280)	Xerophytic rosette shrubland

^**A**^Based on information obtained from CONABIO [16], García [17], Gentry [18], Illsley et. al [19] and Ruiz-Corral et. al [20]. †Mean annual precipitation (mm).

**Figure 5:**
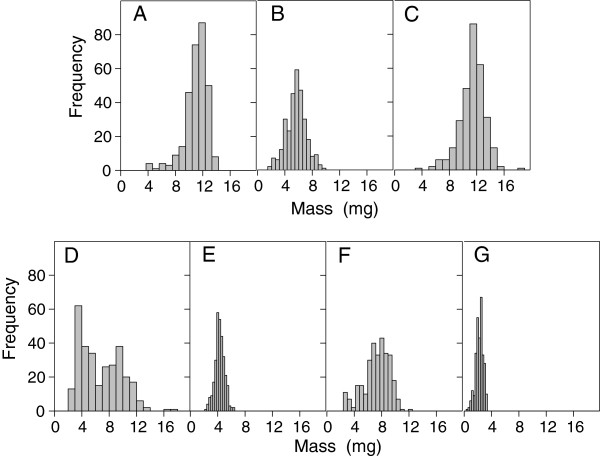
**Frequency distribution of seed size (mass) of** ***Agave*** **spp.** ***n*** **= 300.** **A**; *A. americana* var. *marginata*, **B**; *A. asperrima*, **C**; *A. cupreata*, **D**; *A. duranguensis*, **E**; *A. lechuguilla*, **F**; *A. salmiana* and **G**; *A. striata*.

Seed water uptake is governed by several variables including seed size and biomass [15], seed-soil Ψ_W_ difference, seed contact area as affected by vapour or liquid transfer, and conductive properties of the seed for both liquid and vapour phases, among others [12]. In the present study, seeds experienced similar conditions during germination, except substrate Ψ_W_. Maximum seed water uptake was high in some species with a low seed biomass, but no general correlation between the two variables was noted. *A. asperrima* and *A. striata* had a low mean seed biomass (2 and 5 mg; Figure 5) and the highest maximum seed water uptake (160%), but *A. americana* var. *marginata*, with one of the larger mean seed biomass (11 mg), had the lowest maximum seed water uptake (105%) at -0.03 MPa (Figures 1A, 2B and 5). Differences in maximum seed water uptake among species (Figure 2B) were partly similar to those found by Peña-Valdivia et al. [2] for three *A. salmiana* genotypes with different seed sizes. We suggest that this result might be evidence for differences in seed water permeability among *Agave* species. Different ways of water entry into the seed and contrasting sequences of seed structure imbibition among species and cultivars have been previously documented [21, 22]. Furthermore, differences in maximum seed water uptake during phase II of germination are due to changes in volume and reorganisation of seed macromolecules (e.g. polysaccharides and proteins) [23] that promote more places on seed for water absorption [24]. During this process, changes within the seed occur at different rates. Thus, low water availability (Ψ_W_ between -1.0 and -1.5 MPa) might promote partial hydration and volume increase of macromolecules in *Agave* seeds. Despite controversy regarding the role of seed composition in seed water uptake, heterogeneity in maximum seed water uptake between a barley mutant and a cultivar was explained by differences in seed chemical composition [25]. In this context, Brancalion et al. [26] observed a positive relationship between seed protein content and critical seed water content for germination in five Brazilian tropical woody species.

Differences in maximum seed water uptake during phase II of germination in *Agave* species (Figure 2B) were similar to those for *Festuca rubra* ssp. *litoralis*, *Lolium perenne* and *Poa pratensis*[27]. Results suggest that *Agave* seeds at low substrate Ψ_W_ reached a hydration threshold for radicle emergence. Therefore, more time is required to reach the hydration threshold at low Ψ_W_ and radicle emergence will not occur [12]. The seed water uptake threshold in most *Agave* species was approximately half that reached at the highest Ψ_W_ (Figure 1A-B). This difference was larger than that obtained with seeds of *Lolium perenne* and *P. pratensis*; seed water uptake to germination in these species diminished from 100 and 90% to an asymptotic level of 90 and 80%, respectively, when the Ψ_W_ changed from zero to -0.8 MPa or less [27].

### Radicle emergence

The lack of dormancy in several *Agave* species and genotypes has been documented [1, 2], as well as the high reproductive effort expressed by semelparity and the production of a huge amount of seeds by a single plant [28]. In contrast, most *Agave* species in field conditions predominantly present asexual reproduction [29]. The small seed size, which varied between 2 and 11 mg (Figure 5), in relation to the high plant biomass (200 kg estimated for an *A. salmiana* adult plant), differs from the usual positive correlation found between seed and plant size [30]. However, a lack of dormancy and massive production of small and viable seeds might favour the emergence of a large quantity of seedlings when environmental conditions are favourable, and represent an opportunity for repopulation based on sexual reproduction.

The lowest substrate Ψ_W_ reduced the maximum accumulated germination of some species (p < 0.05) (Figure 3C). *Agave* germination at such restrictive substrate Ψ_W_, under laboratory conditions, might partially mirror the response in the natural environmental conditions where each species grows (Table 2). The low percentage of germination of *A. cupreata* (20%) at -1.5 MPa (Figure 3C) appears to be related to the sub-humid climate (838 mm mean annual precipitation) where this species is distributed and contrasts with the non-significant effect of this low Ψ_W_ on germination of *A. striata* (85%), *A. americana* (78%) and *A. lechuguilla* (70%) and the arid and semi-arid climate (280–580 mm mean annual precipitation) within their natural distribution range. Nevertheless, this relationship was not general, as *A. asperrima* reached a very low final cumulative germination (30%) at Ψ_W_ of -1.5 MPa, despite originating from a semi-arid climate region (450 mm mean annual precipitation).

A lack of germination after 732 h, at -1.5 MPa, suggests that some *Agave* seeds lack some stimulus for germination, despite the imbibition of a similar amount of water as those seeds already germinating at this specific Ψ_W_ (Figures 1 and 2). The lack of germination under optimal conditions has been considered as dormancy [12, 31], and occurs by a variation of the critical seed Ψ_W_ to germinate among individual seeds within the same population. Therefore, whereas the seed Ψ_W_ remains at a partially limiting level, the cumulative germination remains as an asymptote. Inhibition and the absence of germination for some *Agave* species at -1.0 and -1.5 MPa (Figure 3B-C) might be evidence of their tolerance to low substrate Ψ_W_. We recognised -1.5 MPa as an adequate substrate Ψ_W_ to identify the effects of water availability on seed germination of *Agave* spp. and as a stress factor for germination of these species.

The hydrotime model indicates that seeds do not germinate below a Ψ_W_ threshold, but up to this value, seeds germinate because of θ_H_ accumulation. Each seed in a population varies in the threshold level of accumulated θ_H_ necessary to germinate, but θ_H_ is constant for the population as a whole [11]‒[13]. Repeated measures analysis indicated that accumulation of θ_H_ was delayed by the low Ψ_W_. Also it indicated that germination at -1.5 MPa significantly increased at 12, 24 and 36 h intervals; whereas at -0.03 and -1.0 MPa significant increase was observed every 12 or 24 h. Then the Ψ_W_ threshold was lower than -1.0 MPa for the seven species, but even lower for some of them, as *A. striata* and *A. americana* (Figure 3C).

The huge difference in minimum water availability necessary for germination among *Agave* species was demonstrated by the variation in θ_H_ from 137 MPa h for *A. duranguensis* to 282 MPa h for *A. salmiana* (Table 1). According to Bradford [12], θ_H_ is an indicator of seed vigour and physiological quality; results suggested that there was a gradient of vigour among *Agave* species (Table 1). In general, the hydrotime model adequately fitted data for the germination of *Agave* spp. (mean r^2^ = 0.83). However, individual r^2^ values indicated that the hydrotime model better explained seed germination of *A. asperrima* and *A. lechuguilla* than that of *A. duranguensis* and *A. salmiana* (Table 1).

The linear relationship between the rate of *Agave* seed germination and substrate Ψ_W_ (Figure 4) was similar to that observed for sugar beet [11] and *Eurotia lanata* (Pursh) Moq. [32]. The rate of *Agave* seed germination correlated positively with maximum seed water uptake (r = 0.74, p < 0.05); although seeds absorbed more water when water was abundant than when it was restrictive, each seed had a seed water uptake threshold (Ψ_bg_) to initiate germination [13]. The Ψ_b(50)_ of *Agave* species (Table 1) was relatively similar to that of *Solanum lycopersicum* seeds when they were exposed to a low substrate Ψ_W_, which promoted their germination [33] and was also similar to that of a group of 14 coloniser species [15]. In contrast, higher variation in Ψ_b(50)_ (from -0.07 to -5.92 MPa) was reported by Allen et al. [9] for 24 xerophyte species grouped as species adapted to salinity, sandy soils and diverse habits (generalist species). The Ψ_b(50)_ has been considered as an indicator of species adaptation to the environment, since salinity-tolerant plants had the lowest Ψ_b(50)_[9]. The Ψ_b(50)_, of halophytes [9] and those of *A. salmiana*, *A. americana* and *A. striata* (Table 1), suggests that germination can occur in conditions of partly dry soil. According to climate data for the distribution regions (Table 2), *A. striata*, *A. lechuguilla* and *A. salmiana* might have less available water than the other species for germination under natural conditions, because the mean annual rainfalls for their habitats are the lowest (287–361 mm). However, maximum accumulated germination and rate of germination at low substrate Ψ_W_, θ_H_, Ψ_b(50)_ and their dependence on seed mass appears not to be a distinctive characteristic for these species.

Values of Ψ_b(50)_ closer to zero for *A. asperrima* and *A. duranguensis* (Table 1) indicate that germination might only occur under well-watered soil conditions as Allen et al. [9] observed for sandy soil species; however, results in Figure 3C indicate a different trend. We presume that both these species might have adapted to germinate during the wet season. Similar findings were observed for *S. lycopersicum* cultivars, where genetic improvement for tolerance or drought resistance significantly diminished the Ψ_b(50)_, whereas high values for this variable were characteristic for drought-sensitive cultivars [34].

The similar σ_Ψb_ among species indicates some degree of seed uniformity; small differences among *Agave* species in Table 1 might result from partial homogeneity of seed size (mass) among the species, because seeds used in the study were restricted to a seed biomass within one σ range of the total sample of each *Agave* species (Figure 5). The large σ_Ψb_ for *A. salmiana* could be interpreted as a high ecological plasticity, as Ψ_bg_ is more variable, and these seeds might germinate under widely variable water conditions (Figure 3). Germination of *A. salmiana* in extreme environmental conditions, such as high temperature, was recently reported [1].

### Recovery of germination

Seeds of most *Agave* species remained imbibed for more than 30 d with 70% hydration without damage (Figure 3C). The ability of these seeds to remain partially hydrated without germination has been considered as a type of dormancy and appears to be an appropriate response to arid and semi-arid environments, since if germination occurs at very low substrate Ψ_W_ seedling survival will decrease [31]. This seed tolerance appears to be associated with the so-called “seed hydration memory” of several desert Cactaceae by Dubrovsky [35, 36].

All these results confirm that *Agave* seeds can germinate under adverse environment [1, 2] but with some diverse responses to the natural environment among species. It has been reported that sexual reproduction of *Agave* spp. plants is unusual in the wild [28], therefore, it is necessary to study the repopulation process in the field.

## Conclusions

Several *Agave* species lack seed dormancy independently of species distribution. *Agave* species reach about half the maximum seed water uptake under low water availability compared with well-watered substrates and most of them reach more than 50% germination. Several *Agave* species can maintain low seed water uptake for several weeks and germinate rapidly when water availability increases. Germination responses of *Agave* species from dry climate suggest adaptation to low water availability. Seed biomass do not appears to be a distinctive characteristic for responses to water availability during germination.

## Methods

### Plant material

Seven Mexican *Agave* species were studied, which all had ancestral or current economic importance and belonged to different geographical distributions with different climates, and had sexual or asexual (or both) reproduction strategies or diverse systematic positions (Table 2). Fruits from vigorous healthy plants were harvested and dried under laboratory conditions (25°C and low relative humidity). To standardise seed water content, seeds of each species were placed in a cheesecloth-like bag inside a glass jar three months prior to experimentation and maintained at 4 ± 1°C. Subsequently, four replicates, each with five seed, were used to measure seed water content, which was 7.62 ± 0.52%.

### Seed biomass stratification

Seed biomass was obtained from a sample of 300 seeds from each species (Figure 5). Because variation in seed biomass within a species might affect germination, only seeds within one standard deviation of the mean seed biomass for each species were used [1]. *Agave duranguensis* showed a bimodal seed size distribution; a preliminary assay of germination showed little or no germination of lighter seeds, therefore, heavier seeds of this species were used for this study.

### Substrate water potential and experimental conditions

Vermiculite was rinsed in tap water and then dried at 100°C to constant weight. Three different vermiculite Ψ_W_ (-0.03, -1.0 and -1.5 MPa) were obtained by mixing 100 g dry vermiculite with 170, 16 or 11 mL distilled water (w:v) in polyethylene bags. These were sealed for 48 h before the start of the assay, according to Peña-Valdivia and Sánchez-Urdaneta [6], after which time a vermiculite sample was incubated in a psychrometric chamber (Wescor C-52, Inc, Utha, USA) for 4 h, and its Ψ_W_ was subsequently determined by connecting the chambers to a microvoltmeter (Wescor HR-33 T, Inc, Utha, USA) operated in the dew-point mode.

Individual seeds were sown at 1 cm depth in cylindrical polyvinyl chloride (PVC) containers (40 mm diameter, 50 mm depth). Containers were filled with vermiculite at each Ψ_W_ and sealed with a piece of black polyethylene, which was fixed with an elastic band to keep constant Ψ_W_ and were kept in the dark at 25 ± 2°C.

### Data recording and analysis

Seed water uptake (expressed as a percentage of initial seed biomass) and seed germination were recorded every 12 h or daily up to maximum values. Germination was considered to have occurred when the emerging root was at least 5 mm long and maximum germination in each treatment was determined when no additional seeds germinated after 3–4 days. Vermiculite from each experimental unit was renewed every 72 h to maintain a constant substrate Ψ_W_. When treatments at -1.5 MPa had reached maximum germination, non-germinated seeds were transferred to vermiculite at -0.03 MPa and kept until germination occurred or seed damage appeared.

Germination attributes were analysed using the hydrotime model. According to this model [11]‒[13], seed germination can be described using the following equation:


where Ψ_bg_ was the base water potential (MPa) at which the g seed sample germinates, Ψ_W_ was the substrate water potential (MPa), θ_H_ was the hydrotime constant, or the accumulated water-time required to germinate a one g sample of a seed population (MPa h), and t_g_ was the time from water uptake until germination of the g fraction (h or d). It was assumed that Ψ_bg_ was normally distributed within a seed population, and that θ_H_ was constant for all seed fractions [13]. Apart from seed Ψ_bg_, the median population Ψ_bg_ can be estimated (Ψ_b(50)_) and was defined as the base or minimum Ψ_W_ necessary to achieve 50% germination. At this level of germination, it was possible to quantify the time taken for 50% germination (t_50_) or the rate of germination (1/t_50_). The standard deviation (σ_Ψb_) of Ψ_b(50)_ was the parameter of Ψ_bg_ variability. The hydrotime parameters were calculated as described by Allen et al. [9].

### Experimental design and statistical analysis

This study was conducted using a completely randomised design, with a factorial (7 × 3) arrangement of treatments and five experimental units; each unit was a group of 10 PVC containers. Factors were species (*A. americana* var. *marginata, A. asperrima, A. cupreata, A. duranguesis*, *A. lechuguilla*, *A. salmiana* and *A. striata*) and substrate Ψ_W_ (-0.03, -1.0 and -1.5 MPa). The normality test was performed based on graphic residual analysis and the Shapiro-Wilks test for data for initial and maximum seed water uptake, rate of germination (1/t_50_) and the maximum accumulated percentage of germination at every time. Data were transformed when normality supposition failed. Variables were analysed using the GLM procedure of SAS software, 9.17 version (SAS Institute, NC, USA) and significant differences among treatment means were established using Tukey’s honest significant difference (HSD) test with α = 0.05.

A repeated measurements test was performed in order to analyse variation of cumulatove germination along the time. For this analysis Statistica Ver. 6 software was used.
